# Effectiveness of a Theory- and Web-Based Adaptive Implementation Intervention on Nurses’ and Nursing Students’ Intentions to Provide Brief Counseling: Protocol for a Randomized Controlled Trial 

**DOI:** 10.2196/18894

**Published:** 2020-07-31

**Authors:** Guillaume Fontaine, Sylvie Cossette, Marie-Pierre Gagnon, Véronique Dubé, José Côté

**Affiliations:** 1 Research Center Montreal Heart Institute Montréal, QC Canada; 2 Faculty of Nursing Université de Montréal Montréal, QC Canada; 3 Faculty of Nursing Université Laval Québec, QC Canada; 4 Research Center CHU de Québec Québec, QC Canada; 5 Research Center University of Montreal Hospital Center Montréal, QC Canada

**Keywords:** brief counseling, implementation science, knowledge translation, eLearning, clinical practice improvement, nursing education

## Abstract

**Background:**

Brief counseling can motivate patients to initiate health behavior change. However, increasing the provision of brief counseling by nurses is difficult due to contextual and practitioner-level factors impeding nurses’ motivation and intentions to provide brief counseling (eg, unfavorable attitude toward brief counseling, lack of perceived control linked to barriers). Theory-based implementation interventions could address these practitioner-level factors and support evidence-based practice in the context of brief counseling. Web-based, adaptive e-learning (electronic learning) programs are a novel type of implementation intervention that could address the limitations of current brief counseling training programs, such as accessibility and personalization.

**Objective:**

This paper presents a study protocol for evaluating the effectiveness of the E_MOTIV_A_ implementation intervention—a theory- and web-based adaptive e-learning program—to increase nurses’ and nursing students’ intentions to provide brief counseling for smoking, an unbalanced diet, and medication nonadherence.

**Methods:**

A two-group, single-blind, randomized controlled trial will be conducted with nurses and nursing students enrolled in a Bachelor of Science in Nursing program in Quebec, Canada. Participants in the experimental group will be allocated to the E_MOTIV_A_ intervention—a theory- and web-based adaptive e-learning program—while participants in the active control group will be allocated to the E_MOTIV_B_ intervention, a knowledge- and web-based standardized e-learning program. The E_MOTIV_A_ intervention was designed to influence the constructs of the Theory of Planned Behavior (eg, attitude, subjective norms, and perceived behavioral control) in the context of brief counseling. The Cognitive Load Index and User Engagement Scale will be used to assess participants’ cognitive load and engagement related to e-learning. Participants will complete the Brief Counseling Nursing Practices Questionnaire–Abridged Version at baseline and follow-up. All study measures will be completed online.

**Results:**

The study is ongoing. The results of the study will provide answers to the primary hypothesis (H1) that experimental group participants will demonstrate a greater change in the score of intentions to provide brief counseling between baseline (–T1) and follow-up (T4). Secondary hypotheses include greater improvements in scores of attitude (H2), subjective norms (H3), perceived control (H4), behavioral beliefs (H5), normative beliefs (H6), and control beliefs (H7) regarding brief counseling in the experimental group between baseline and follow-up. We also anticipate lower intrinsic and extrinsic cognitive loads (H8, H9), higher germane cognitive load (H10), and higher engagement (H11, H12) in the experimental group.

**Conclusions:**

This study will be among the first in evaluating a novel type of implementation intervention, a theory- and web-based adaptive e-learning program, in nurses and nursing students. This type of intervention has the potential to support evidence-based practice through accessible, personalized training in wide-ranging domains in nursing.

**Trial Registration:**

ISRCTN Registry ISRCTN32603572; http://www.isrctn.com/ISRCTN32603572

**International Registered Report Identifier (IRRID):**

PRR1-10.2196/18894

## Introduction

### Noncommunicable Diseases and Behavioral Risk Factors

Noncommunicable diseases, including cardiovascular diseases, diabetes, cancers, and chronic respiratory diseases, are major contributors to global morbidity and mortality [1]. In Canada, noncommunicable diseases were responsible for 88% of deaths in 2017 [1]. These diseases are caused mainly by behavioral risk factors, such as smoking, an unbalanced diet, and medication nonadherence [2]. The prevalence of smoking among Canadians was 17.9% in 2014 [3]. Through hemodynamic, hemostatic, and inflammatory mechanisms, smoking leads, on average, to a life expectancy of 10 years lower [2]. The prevalence of an unbalanced diet among Canadians, defined as a failure to meet the fruit and vegetable consumption threshold according to Canada’s food guide [4], was 60.3% in 2014 [3]. Diet plays an important role in the pathophysiology associated with noncommunicable diseases through several mechanisms of action [2]. Finally, while medication adherence varies by population and context, a meta-analysis highlighted that 40% of patients do not take their recommended treatment for their cardiovascular problem (defined by adherence 80%) [5]. Thus, these behavioral risk factors should be targeted by interventions initiated by health care professionals, including nurses [6].

### Brief Behavior Change Counseling

When provided by trained health care professionals, motivational approaches such as brief behavior change counseling, hereafter called brief counseling, can help patients initiate and maintain health behavior change [6]. Brief counseling, lasting from 1 to 15 minutes, aims to explore the individual’s motivation and capabilities and to intervene to encourage and support behavior change [7-9]. Brief counseling is associated with modest but clinically significant effects for smoking cessation, the adoption of a balanced diet, and medication adherence [10,11].

Nurses and nursing students are well-positioned across the continuum of care to provide brief counseling to explore patients’ beliefs, assess their level of motivation for change, and intervene to elicit behavior change [12]. However, brief counseling requires specific knowledge and skills [12]. While nursing students acquire foundational knowledge about health communication and develop relational skills (eg, active listening, reformulation), training does not generally cover specific abilities to explore patients’ motivation and ambivalence and to intervene accordingly. In addition, nurses and nursing students’ attitudes toward brief counseling can be variable and may alter their motivation in providing brief counseling in different care settings. Some may believe these settings are not conducive to brief counseling and health behavior change [13].

Similarly, the subjective norm (ie, the perceived social pressure onwards) toward brief counseling is influenced by the attitudes and behaviors of nurses and other professionals [13]. There is also a need to increase nurses’ and nursing students’ perceived behavioral control over brief counseling by addressing barriers, highlighting facilitators, and providing knowledge and skills to intervene [14]. According to the Theory of Planned Behavior (TPB), these sociocognitive determinants (ie, attitude, subjective norm, perceived behavioral control) are predictors of nurses’ and nursing students’ intentions to provide brief counseling, and of its actual provision in clinical practice [15].

### Theory-Based Implementation Interventions

Sociocognitive determinants may be used for designing a theory-based *implementation intervention* to increase the provision of brief counseling by nurses and nursing students [16]. An implementation intervention is defined as any strategy or program “aimed at increasing the use of research-based knowledge in healthcare practice (pg 2)” [17]. Examples of these interventions, sometimes called “implementation strategies,” including audit and feedback, educational materials, e-learning (electronic learning), educational games, communities of practice, local opinion leaders, and reminders [18]. Practitioner-level implementation interventions target behavior change at the level of individual health care professionals and teams (ie, nurses and nursing students in this context) [17]. These interventions may be based on a wide range of theories, models, and frameworks, including theories of behavior and behavior change, such as the TPB [19]. Studies that evaluate implementation interventions based on the TPB have become more common in recent years [20-22]. For example, a study by Welch [22] used the TPB to design an implementation intervention, a web-based e-learning program, aiming to influence the moral norm, or sense of professional responsibility, to promote the uptake of brief counseling by nurses. Additional studies are warranted to solidify the evidence base [16,21].

### Adaptive e-Learning Programs

Studies have shown the benefits of e-learning and the use of technology to support learning and clinical practice change [23,24]. Recently, adaptive e-learning has emerged has a novel type of practitioner-level implementation intervention [24]. Adaptive e-learning programs collect data at different points during a training program (eg, attitude of each learner) to determine, from multiple pathways conceptualized by a team of experts or by computer algorithms, the optimal learning path for each learner [24]. Adaptive e-learning programs mimic face-to-face learner-teacher interactions, where the teacher adapts learning content and format based on learners’ feedback. Adaptive e-learning programs could alleviate the current limitations of most brief counseling training programs. Indeed, most brief counseling training programs assessed with nurses and nursing students have been mainly face-to-face, limiting accessibility, and group-based, reducing personalization [12].

Two main cognitive processes have been studied to optimize human-computer interaction in the context of adaptive e-learning: engagement and cognitive load. Engagement, the learner’s investment when interacting with an e-learning program, should be maximized [25]. The cognitive load refers to how much the learner’s working memory is solicited during learning [26]. Three types of cognitive loads have different effects on learning: 1) intrinsic load, linked to the complexity of the learning task, should be adapted to the learner; 2) extrinsic load, linked to superfluous or confusing elements during learning, should be minimized; 3) germane load, linked to the integration of the programs’ concepts, should be maximized. Considering learners’ engagement and cognitive load is crucial when developing and evaluating an adaptive e-learning program. More specifically, considering engagement is important in this context since giving learners control over their learning path in an adaptive e-learning program may increase their engagement. In addition, personalizing instruction through data collected at multiple time points during the training may also increase the learner’s engagement in the e-learning program. Regarding cognitive load, it is hypothesized that personalizing instruction in an adaptive e-learning program may reduce extrinsic load and increase germane load [24].

To our knowledge, only one prior study evaluated a theory- and web-based adaptive e-learning program targeting sociocognitive determinants to support the provision of brief counseling by nurses, and none was conducted with nursing students. The study was conducted in The Netherlands to support the provision of brief counseling by primary care nurses for smoking cessation [21]. Results showed that the e-learning program increased the provision of brief counseling for smoking cessation among a subset of these nurses [21]. Thus, this paper adds to the emerging literature by presenting a protocol for evaluating the E_MOTIV_A_ implementation intervention—a theory- and web-based adaptive e-learning program—to increase nurses’ and nursing students’ intentions to provide brief counseling.

### Study Objective and Hypotheses

The objective of this randomized controlled trial (RCT) is to evaluate the effect of a theory- and web-based adaptive e-learning program targeting the constructs of the TPB (E_MOTIV_A_ intervention; experimental group), versus a knowledge- and web-based standardized e-learning program (E_MOTIV_B_ intervention; active control group), on nurses’ and nursing students’ intentions to provide brief counseling for smoking cessation, the adoption of a balanced diet and medication adherence. Our primary hypothesis (H1) is that experimental group participants will demonstrate a greater change in the score of intentions to provide brief counseling between baseline (–T1) and follow-up (T4).

Secondary hypotheses include greater improvements in scores of attitude (H2), subjective norm (H3), perceived behavioral control (H4), behavioral beliefs (H5), normative beliefs (H6), and control beliefs (H7) regarding brief counseling in the experimental group between baseline and follow-up. We also anticipate lower intrinsic and extrinsic cognitive loads (H8, H9), higher germane cognitive load (H10), and higher experiential and behavioral engagement (H11, H12) in experimental group participants compared with control group participants.

We will also explore links between theoretical constructs of the study model—ie, what sociocognitive determinants at baseline (–T1) are correlated with the intentions to provide brief counseling at the follow-up (T4).

## Methods

### Trial Design

A two-group, single-blind, parallel RCT will be conducted to evaluate the effectiveness of the E_MOTIV_A_ experimental intervention compared to the E_MOTIV_B_ control intervention. This protocol is presented according to the Standard Protocol Items: Recommendations for Interventional Trials (SPIRIT) 2013 guideline [27]. The Consolidated Standards of Reporting Trials (CONSORT) eHEALTH checklist is presented in Multimedia Appendix 1. The protocol was prospectively registered on October 14, 2019 (ISRCTN32603572).

### Study Setting and Eligibility Criteria

The study will be conducted entirely online at a Faculty of Nursing in a major university in Quebec, Canada. In the province of Quebec, nurses can practice with a 3-year College Diploma in Nursing, or with a 3-year Bachelor of Science in Nursing (BSN) degree achieved at the university level. However, nurses with a College Diploma can still pursue university-level education with a shorter, 2-year BSN program. Thus, BSN programs in Quebec include both nurses and nursing students. For this reason, while the study will be conducted in a BSN program, it targets both nurses and nursing students (hereafter called “participants”).

The inclusion criteria are: (1) to be a BSN student in a primary health care course; (2) to be able to perform computer tasks (eg, taking emails); (3) to understand French. There is no exclusion criterion.

### Interventions

This section provides a high-level summary of the interventions. An in-depth description of both interventions will be published in a forthcoming paper.

#### Experimental Group (E_MOTIV_A_)

**Figure 1:**
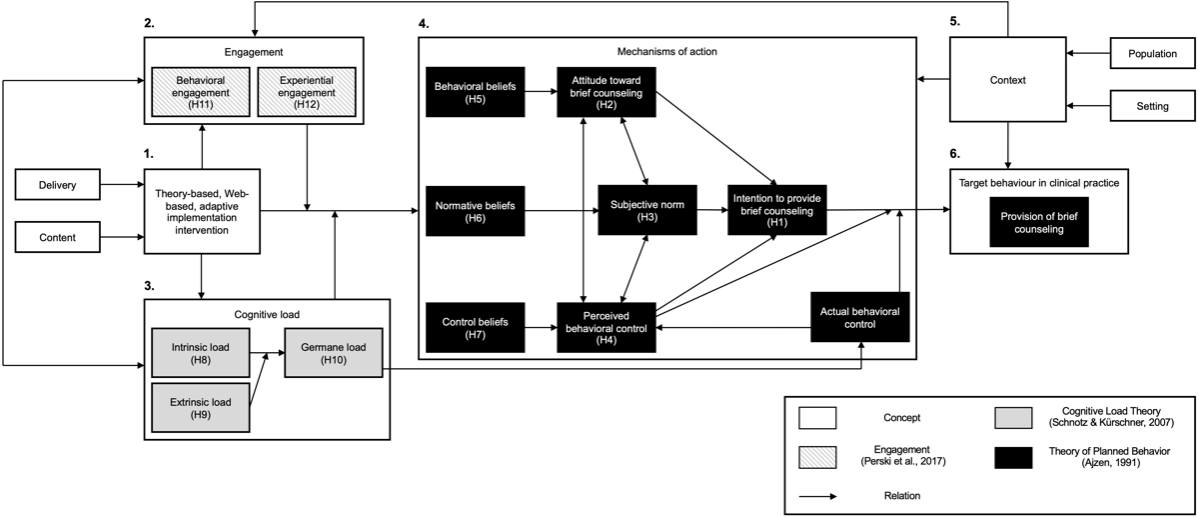
The integrative theoretical framework of the study, based on the Theory of Planned Behavior [15], Cognitive Load Theory [29], and the concept of engagement with digital interventions [32].

Participants (ie, nurses and nursing students) in the experimental group will receive access to the “E_MOTIV_A_ intervention,” a web- and theory-based adaptive e-learning program incorporating learning content delivered through text, pictures, and videos on (1) smoking, unbalanced diet, medication nonadherence; (2) treatment options; and (3) brief counseling (Table 1). This content was piloted with 31 nurses [9,28]. Moreover, the E_MOTIV_A_ intervention incorporates content based on an integrative theoretical framework including (1) the TPB [15]; (2) Cognitive Load Theory [29]; (3) the concept of engagement [25] (Figure 1). The TPB posits that *sociocognitive determinants* (ie, attitude, subjective norms, and perceived behavioral control) influence participants’ *intentions*, which in turn, with *actual behavioral control* (eg, external factors), influences participants’ *behavior* in clinical practice. The second component is the Cognitive Load Theory, which describes principles aimed at linking the E_MOTIV_A_ intervention to the cognitive architecture of learners. The third component is the concept of engagement, acting as a mediator between the E_MOTIV_A_ intervention and its effects on sociocognitive determinants in participants. The E_MOTIV_A_ intervention targets 7 sociocognitive determinants amenable to change in participants regarding brief counseling (Table 1). To change these determinants and increase participants’ intentions to provide brief counseling in clinical practice, the E_MOTIV_A_ program incorporates 19 strategies (ie, behavior change techniques) (Table 1). The e-learning program was developed with a web agency near Montreal, Canada.

More specifically, E_MOTIV_A_ consists of 3 adaptive training sessions. Each session includes a fixed number of “navigation adaptation points” consisting of questions asked to participants. There are two types of these adaptation points:

1. *Suggested* navigation adaptation points, where the participant can determine the preferred learning path from multiple options. This is operationalized as a question asked to participants, for example, “Which cardiovascular risk factor do you wish to see first in this training program from the options presented below?” The participant can then select one of three options (smoking, unhealthy eating habits, medication nonadherence), and the platform will dynamically adapt the learning path.
2. *Enforced* navigation adaptation points, where the participant answers a question with a 4-point response scale (agree, agree slightly, disagree slightly, disagree), for example, “Helping patients change their health behaviors (like smoking) is complex.” Depending on their answers, participants are automatically sent to different learning paths. In this specific example, if the participant answers “Agree,” “Agree slightly” or “Disagree slightly,” they are sent to a video of a nurse practitioner explaining that while health behavior change can be complex and difficult, it can also often be spontaneous and does not necessarily involve a long process (eg, quitting smoking).

The first training session focuses on noncommunicable diseases, cardiovascular risk factors, the foundations of health behavior change, and the 5 As brief counseling approach. It includes three navigation adaptation points: (1) one focusing on cardiovascular risk factors; (2) one focusing on the participant’s beliefs about brief counseling (eg, the effectiveness of brief counseling); and (3) one focusing on the participant’s perceived ability or control over the provision of brief counseling (eg, how much time it takes to provide brief counseling). The second training session focuses on strategies and resources for smoking cessation, the adoption of a balanced diet and medication adherence, as well as on multiple role-playing videos of brief counseling with a nurse and patients presenting different levels of motivation toward behavior change. It includes four navigation adaptation points: (1) one focusing on normative beliefs about brief counseling (eg, what do doctors think about brief counseling); (2) one focusing on role-playing videos of brief counseling with unmotivated patients; (3) one focusing on role-playing videos of brief counseling with motivated patients; and (4) one focusing on participants’ intention to provide brief counseling. The third training session allows students to review any content from previous sessions but does not include new content.

##### Control Group (E_MOTIV_B_)

Participants in the control group will access the “E_MOTIV_B_ intervention,” a knowledge- and web-based standardized e-learning program (Table 1), including learning content on risk factors, treatment options, and brief counseling. The E_MOTIV_B_ intervention targets primarily 2 sociocognitive determinants in participants regarding brief counseling. However, the intervention may also affect other sociocognitive determinants (eg, attitude, behavioral beliefs). To change these determinants, E_MOTIV_B_ includes 6 strategies frequently used in nursing education [31].

**Table 1:** A high-level description of the E_MOTIV_A_ and E_MOTIV_B_ interventions.

Characteristic		E_MOTIV_A_ intervention (experimental group)	E_MOTIV_B_ intervention (control group)
Description		A theory- and web-based adaptive e-learning program focusing on brief counseling	A knowledge- and web-based standardized e-learning program focusing on brief counseling
**Training Sessions**			
	Number of sessions	3	3
	Duration of each session	Session duration will vary in function of the learning paths. Session 1: ~50 min Session 2: ~60 min Session 3: ~20 min	Session duration is fixed for sessions 1 and 2, and variable for session 3. Session 1: ~40 min Session 2: ~50 min Session 3: ~20 min
Clinical focus of the e-learning program		Brief counseling for smoking, unbalanced diet and medication nonadherence	Brief counseling for smoking, unbalanced diet and medication nonadherence
Brief counseling approach taught		5 As (Ask-Assess-Advise-Agree-Assist)	5 As (Ask-Assess-Advise-Agree-Assist)
Sociocognitive determinants targeted by the training program in participants		The E_MOTIV_A_ intervention is personalized to 7 sociocognitive determinants in participants to increase the provision of brief counseling in clinical practice Intention Attitude Subjective norm Perceived behavioral control (eg, knowledge, skills) Behavioral beliefs Normative beliefs Control beliefs	The E_MOTIV_B_ intervention targets primarily 2 sociocognitive determinants in participants to increase the provision of brief counseling in clinical practice Perceived behavioral control (eg, knowledge, skills) Control beliefs To a lesser extent, the intervention may affect other sociocognitive determinants.
Strategies (ie, behavior change techniques) to change the sociocognitive determinants in participants		To increase participants’ intentions to provide brief counseling in clinical practice, the E_MOTIV_A_ intervention incorporates 19 strategies, including the 6 of the E_MOTIV_B_ program. Examples of these strategies include Encouraging the substitution of existing practices (information-giving) for brief counseling. Providing information on the approval of other care team members regarding providing brief counseling.	The E_MOTIV_B_ intervention includes 6 strategies frequently used in nursing continuing education Role modeling, ie, the demonstration of brief counseling skills by an expert nurse with patients in videotaped simulated clinical encounters. Instructions on how to provide brief counseling for smoking, unbalanced diet, and medication nonadherence.

### Variables, Measures, and Data Collection

Study variables and measures are presented in Table 2. First, a 15-item sociodemographic questionnaire will be completed at the baseline. Second, we will use the Brief Counseling Nursing Practices Questionnaire Abridged Version (BCNPQ–AV) at baseline [33] and at the follow-up to measure nurses’ intentions (H1), and other sociocognitive determinants regarding brief counseling (H2-H7). The BCNPQ was developed originally in French based on the TPB [33]. The BCNPQ–AV has 7 subscales and 48 items. Items have an 8-point (0-7) Likert-type response scale. Third, after two training sessions, we will use the French version of the Cognitive Load Index (CLI) [34] to measure participants’ cognitive load related to the e-learning programs. The 10-item French version of the CLI measures 3 types of cognitive load. All items have an 11-point (0-10) Likert response scale. Mid-range intrinsic load scores, low extrinsic load scores, and high germane load scores are desired. Fourth, we will use the French version of the User Engagement Scale–Short Form (UES–SF) [35] to measure participants’ experiential engagement with e-learning programs. The French version of the UES–SF measures four dimensions of experiential engagement with the e-learning program: (1) focused attention, (2) perceived usability, (3) aesthetic appeal, and (4) reward. All items have a 6-point (0-5) Likert-type response scale. The higher scores are, the more users are engaged with the e-learning program. Psychometric properties of the French versions of the CLI and the UES–SF tested in 57 nursing students [36]. Finally, we will collect usage data through the E_MOTIV_A_ and E_MOTIV_B_ programs. We will collect the duration of use (minutes), the frequency of use (number of logins per user), and the percentage of participants who complete training sessions and who consult each page.

**Table 2:** Study variables and measures.

Study variable		Definition	Instrument/measure	Items, n	Sample question	Internal consistency (α)
Sociodemographic and professional characteristics		N/A^a^	Study-specific questionnaire, self-administered online	15	“How many online courses have you taken in the past?”	N/A
**Sociocognitive determinants**						
	H1: Intention to provide brief counseling	General disposition of the participant to provide brief counseling for smoking, unbalanced diet, and medication nonadherence.	Brief Counseling Nursing Practices Questionnaire—Abridged Version (BCNPQ—AV), self-administered online	15	“Over the next few months, I have the intention to provide brief counseling to my smoking patients.”	.92
	H2: Attitude toward behavior	Latent disposition toward brief counseling based on their emotional response to it and their evaluation of its consequences.	BCNPQ—AV	6	“For me, it is important to provide brief counseling to my patients.”	.81
	H3: Subjective norms	Perceived social pressure toward brief counseling, as a function of the behavior of others (eg, other team members) in the environment.	BCNPQ—AV	4	“Nursing managers believe that I should provide brief counseling to my patients.”	.89
	H4: Perceived behavioral control	Perceived degree of control (capability, opportunity) over the integration of brief counseling in clinical practice.	BCNPQ—AV	7	“I have the skills required to help patients initiate change for the reduction of a cardiovascular risk factor.”	.70
	H5: Behavioral beliefs	Subjective probability of perceiving that brief counseling has favorable or unfavorable attributes.	BCNPQ—AV	4	“If I provided brief counseling, it would make my patients aware of the consequences of cardiovascular risk factors (examples: smoking, poor diet) on their health.”	.84
	H6: Normative beliefs	Subjective probability of perceiving positively or negatively the attitude/behavior of others regarding brief counseling.	BCNPQ—AV	6	“Doctors would disapprove/approve of the fact that I provide brief counseling to my patients.”	.84
	H7: Control beliefs	Subjective probability of considering having the capability and opportunity necessary to perform brief counseling based on the facilitators and barriers.	BCNPQ—AV	5	“I will have the support of my nursing team members to provide brief counseling to my patients.”	.74
**Cognitive load**						
	H8: Intrinsic cognitive load	Cognitive load associated with the task and the learning content.	French version of the Cognitive Load Index (CLI), self-administered online	3	“The subject(s) covered during this activity were very complex.”	.83
	H9: Extrinsic cognitive load	Cognitive load associated with superfluous, unnecessary, or confusing elements are added to the learning task.	CLI	3	“The directions or explanations were ineffective for my learning.”	.70
	H10: Germane cognitive load	Cognitive load reflecting the understanding and integration of the programs’ concepts.	CLI	4	“The activity really improved my understanding of the subject(s) covered.”	.96
**Engagement**						
	H11: Experiential engagement	Subjective experience that emerges from interaction with the e-learning^b^ program characterized by attention, interest, and affect.	French version of the User Engagement Scale—Short Form (UES—SF), self-administered online	12	“The EMOTIV platform was visually pleasing.”	.76 to .89
	H12: Behavioral engagement with e-learning programs	Objective measure of the use of the e-learning program characterized by the number, duration, and period of contacts.	Usage data collected in both e-learning programs	N/A	N/A	N/A

^a^N/A: not applicable.

^b^e-learning: electronic learning.

### Timeline and Procedures

**Figure 2:**
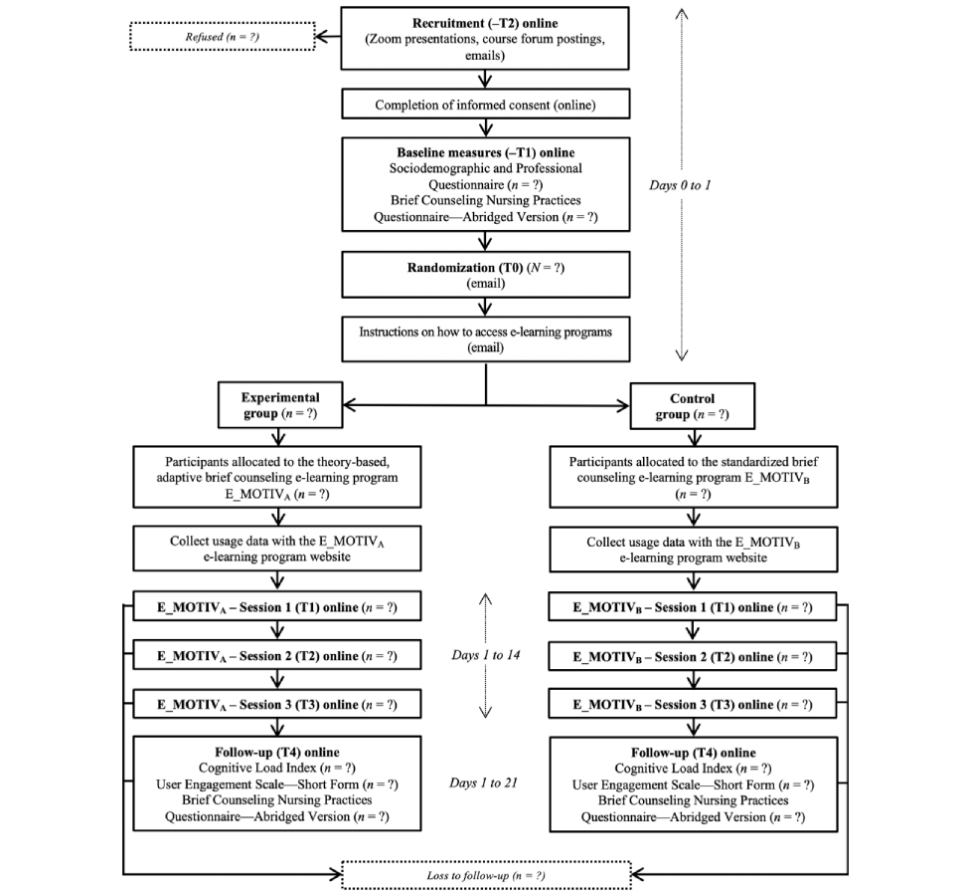
Participant flow diagram.

All study procedures are identical in both study groups, apart from the experimental and control e-learning programs which have different structures, contents, and durations (Figure 2). Participants will be enrolled in the study for up to 21 days, from recruitment (–T2) to follow-up (T4) (Table 3). We estimate that it will take approximately two and a half hours for participants in both groups to participate in the study. After consenting to participate in the study (–T2), participants will immediately be redirected to the online questionnaire to complete baseline measures (–T1). Within 24 hours of completing the baseline measures, participants will be randomized (T0). They will receive an email containing a link to the E_MOTIV_A_ or E_MOTIV_B_ intervention, as well as their user code and password to access the e-learning program. Training sessions 1 (T1), 2 (T2), and 3 (T3) will be completed on the E_MOTIV_A_ or E_MOTIV_B_ web-based platforms within 14 days following randomization. If participants do not want to complete the optional session 3, they will be able to complete the follow-up (T4) measures (ie, the CLI, UES–SF, and BCNPQ–AV online questionnaires) immediately after session 2 by clicking on an embedded link at the end of the session. Otherwise, once participants have completed session 3, they will receive an email containing a link to the follow-up (T4) online questionnaires. Participants will be able to complete follow-up measures until 21 days postrandomization.

**Table 3:** Study timeline.

Activity		Items, n		Day 0				Days 0 to 1		Days 1 to 14						Days 1 to 21	
				Recruitment (−T2)		Baseline measures (−T1)		Randomization (T0)		Training session 1 (T1)		Training session 2 (T2)		Training session 3 (T3, optional)		Follow-up (T4)	
**Recruitment and randomization**																	
	Eligibility evaluation				✓												
	Log of the selection procedure				✓												
	Informed consent				✓												
	Randomization								✓								
	Instructions for training sessions (access)								✓								
**Training sessions**																	
	Access to the E_MOTIV_A_ intervention (experimental group) or E_MOTIV_B_ intervention (control group)										✓		✓		✓		
**Measures collected with online questionnaires**																	
	Sociodemographic questionnaire		15				✓										
	H1 to H7—Brief Counseling Nursing Practices Questionnaire—Abridged Version		48				✓										✓
	H8 to H10—Cognitive Load Index		10														✓
	H11—User Engagement Scale—Short Form		12														✓
**Collected with the e-learning^a^** **programs**																	
	H12—Usage data (eg, frequency, duration)										✓		✓		✓		

^a^e-learning: electronic learning.

### Sample Size and Recruitment

This study seeks to enroll 25 participants per group (BSN students, including nurses and nursing students), for a total of 50 participants (0.75 power; 0.05 bilateral significance level). The calculation is based on the comparison of the change in *intentions to provide brief counseling* (ie, H1; follow-up [T4] score minus the baseline score [−T1]) between the experimental and control groups. We estimate that the standard deviation of the change in intentions will be 6.5. This sample size will allow us to detect a difference of 5 in the score of intentions to provide brief counseling between the two groups. Given the context of the study, carried out as part of a Bachelor of Science in Nursing course, we will not refuse participants once the N is reached to offer all students equal opportunity.

Participants will be recruited through two large group Zoom presentations, course forum postings, and email invitations.

### Randomization and Allocation

The randomization scheme will be generated by the Offsite Coordinating Center (the Montreal Health Innovations Coordinating Center, MHICC). Random assignment will follow a 1:1 allocation with random block sizes to minimize group imbalances.

### Blinding

Both interventions, ie, both e-learning programs, will be completed individually and have the same appearance, name, and main contents to increase the blinding of participants to group allocation. The E_MOTIV_A_ intervention being adaptive, participants will have different learning pathways in that group. This variability in the content and pathways in half of the participants will attenuate contamination between groups if participants discuss their learning experience. The Study Coordinator will be aware of group assignment to (1) create accounts on the E_MOTIV web-based platform for each participant and (2) assign each participant to the experimental or control e-learning program in the E_MOTIV web-based platform.

### Data Analysis

Study variables will be presented by group. The mean, standard deviation, median, minimum, and maximum will be presented for continuous variables, while categorical variables will be described as frequencies and percentages. All statistical tests will be bilateral and with a 0.05 significance level. The Statistical Package for the Social Sciences version 25 will be used to produce intention-to-treat analyses (ie, analysis of all participant data, regardless of study completion) under the supervision of the MHICC.

For the primary outcome, the change in the score of intentions to provide brief counseling (T4-−T1) will be analyzed with a covariance model (ANCOVA), including the group factor and the intentions score at baseline (−T1). This model will allow a comparison of the adjusted mean change in participants’ intentions to provide brief counseling between groups.

Continuous secondary outcomes measured in terms of change between baseline and follow-up (H2 to H7) will be analyzed similarly to the primary outcome. Continuous secondary outcomes measured at follow-up (H9 to H12) will be compared between groups using Student *t* tests or Mann-Whitney tests if variables are not normally distributed.

In terms of exploratory analyzes, the associations between sociocognitive determinants at baseline (−T1) and intentions (H1) will be evaluated using Pearson coefficients or with Spearman coefficients if data are not normally distributed. Multivariate models may be used if data are suitable.

### Ethical Considerations

This protocol has been approved by the University of Montreal Science and Health Research Ethics Board (#20-052-CERSES-D).

## Results

Participant recruitment and enrollment began in spring 2020. Analysis of study results is expected in the summer of 2020 at the end of data collection.

## Discussion

This paper describes a study protocol for evaluating the effectiveness of a theory- and web-based adaptive e-learning program on nursing students’ and nurses’ intentions to provide brief counseling.The E_MOTIV_A_ intervention, which is one of the first of its kind, has important implications for both research and practice. In terms of research, the E_MOTIV_A_ program could be modified to train nurses and nursing students in a wide range of clinical domains. The navigation adaptation points could focus on additional psychological and social constructs for other clinical practices, such as physical assessment, and test ordering. Moreover, additional studies could be conducted to evaluate the E_MOTIV_A_ program, which is an entirely digital intervention, paired with co-interventions implemented directly in care settings, such as local opinion leaders or academic detailing. In terms of practice, E_MOTIV_A_ has the potential to increase the effectiveness and efficiency of learning in nurses and nursing students. Through the adaptivity process, the program can account for the particularities inherent to each learner and provide personalized instruction, potentially increasing engagement and reducing cognitive load.

We remark on two limitations of the present study. First, participants will be randomized individually to the experimental and control groups. Thus, participants in both groups may discuss the project among themselves. Participants will be blinded to group assignment to minimize the risk of contamination. Second, we anticipate that participant retention may present a challenge, given the 10% dropout rate observed with a shorter training program in a previous study. To maximize retention, we will send up to three standardized email reminders will be sent to participants at each study time point (T1, T2, T3, and T4). For example, the reminder for completing the first training session will read as follows: “This email is only a brief courtesy reminder that you can start the first session of the E_MOTIV training program now. You can also start the second training session as soon as you have time. Here is a reminder of the information to log in: […]”.

In conclusion, this study will be among the first in evaluating a theory- and web-based adaptive e-learning program in nurses and nursing students. These programs have the potential to support evidence-based practice through accessible, personalized training in wide-ranging domains in nursing [32,37].

## Appendix

Multimedia Appendix 1 CONSORT-eHEALTH Checklist (V 1.6.1).

## Abbreviations

BCNPQ: Brief Counseling Nursing Practices Questionnaire
BCNPQ–AV: Brief Counseling Nursing Practices Questionnaire–Abridged Version
CLI: Cognitive Load Index
CONSORT–eHEALTH: Consolidated Standards of Reporting Trials E-Health
e-learning: electronic learning
MHICC: Montreal Health Innovations Coordinating Center
RCT: randomized controlled trial
SPIRIT: Standard Protocol Items: Recommendations for Interventional Trials
TPB: Theory of Planned Behavior
UES–SF: User Engagement Scale—Short Form
